# Endoscopic intraventricular hematoma evacuation surgery versus external ventricular drainage for the treatment of patients with moderate to severe intraventricular hemorrhage: a multicenter, randomized, controlled trial

**DOI:** 10.1186/s13063-020-04560-3

**Published:** 2020-07-13

**Authors:** Junhao Zhu, Chao Tang, Zixiang Cong, Jin Yang, Xiangming Cai, Chiyuan Ma, Yuxiu Liu

**Affiliations:** 1 Jinling Hospital, School of Medicine, Nanjing Medical University, 104 Hanzhong Road, Nanjing, 210002 China; 2 Department of Neurosurgery, Jinling Hospital, School of Medicine, Nanjing University, Nanjing, 210002 China; 3 School of Medicine, Southeast University, Nanjing, 210009 China; 4 Department of Medical Statistics, Jinling Hospital, Nanjing Medical University, Nanjing, 210002 China

**Keywords:** Endoscopic surgery, External ventricular drainage, Intraventricular hemorrhage

## Abstract

**Background:**

The application of neuroendoscopy in intraventricular hemorrhage (IVH) has attracted more and more attention in recent years. Studies have shown that the use of neuroendoscopy for IVH evacuation has advantages over external ventricular drainage (EVD) alone. However, the cases of most current research are small and all of them are retrospective studies. The aim of this study is to explore the prognosis of patients with moderate to severe IVH who undergo endoscopic IVH evacuation surgery versus those who undergo EVD alone.

**Methods:**

The study is a prospective, randomized, controlled, multi-center clinical trial. Nine hundred and fifty-six subjects with moderate to severe IVH across four tertiary hospitals in China will be randomly assigned (1:1) to receive either endoscopic IVH evacuation surgery or EVD. The primary objective is to compare patients’ survival rate at 12 months after surgery.

**Discussion:**

The trial is designed to investigate the prognostic benefits of endoscopic IVH evacuation surgery for patients with moderate to severe IVH. Currently, it has never been investigated in a prospective randomized controlled clinical trial.

**Trial registration:**

ClinicalTrials.gov, NCT04037267. Registered on 26 July 2019.

## Background

Spontaneous intraventricular hemorrhage (IVH) is defined as eruption of blood into the cerebral ventricular system caused by rupture of brain arteries, veins, and capillaries instead of trauma. The mortality rate of IVH varies from 50 to 80% in the absence of specific treatment [1].

Primary IVH is confined to the ventricles while most IVH is secondary to intracranial hemorrhages (ICH) involving the thalamus and basal ganglia [2]. IVH is an independent predictor for poor outcome of patients with ICH [3]. According to the results of the STICH trial [4–6], the prognosis of patients with IVH is worse than that of patients without IVH (*p* < 0.00001) and if IVH is associated with hydrocephalus, the prognosis will be the worst.

Although external ventricular drainage (EVD) without thrombolytic agents is firstly used to help drain blood from the ventricles, using EVD alone may not effectively improve the patients’ outcome because of the slow rate of removing intraventricular blood [7]. With increasing clinical studies reporting that intraventricular administration of thrombolytic agents may reduce mortality by accelerating clot lysis [8–10], there has been more interest in using thrombolytic agents as adjuncts to EVD in the setting of IVH. A meta-analysis of 8 observational and 4 randomized studies of patients with IVH treated with EVD (*n* = 149) or EVD with fibrinolysis (*n* = 167) found a significant decrease in mortality from 47 to 23% (OR, 0.32; 95% CI, 0.19–0.52) [11]. Thus, in 2015 AHA/ASA spontaneous cerebral hemorrhage diagnosis and treatment guidelines [12], EVD plus recombinant tissue-type plasminogen activator (rtPA) in IVH patients is suggested, which has a fairly low complication rate; however, the efficacy and safety of this treatment are uncertain (Class IIb; Level of Evidence B).

In 2017, the phase 3 randomized CLEAR III trial, which is the largest trial to test whether using alteplase in removing intraventricular hemorrhage improves functional outcome compared with the placebo (saline), showed that the primary outcome (good functional outcome defined as a modified Rankin Scale score of 3 or less at 180 days) was similar in two groups (alteplase group 48% vs control group 45%; RR, 1.06 [95% CI, 0.88–1.28; *p* = 0.554]) [13]. Although the CLEAR III trial failed to reveal the superiority of thrombolytic agents in its primary outcome, the treatment group had a lower mortality rate (alteplase 46 [18%] vs saline 73 [29%], *p* = 0·006), which is a favorable evidence for making clinical decisions [13].

Several drawbacks of EVD with using thrombolytic agents (mentioned as EVD below) are also found in clinical practice, and the major one is that the catheters need to be removed or replaced regularly which increases the risk of infection.

Alternative procedures have also been reported recently and the endoscopic surgery appears to be the most promising treatment [1, 14–20]. Compared with the microscopic surgery, the endoscopic surgery provides better illustration and close-up view of the operative field, which makes the surgery safer and reduces surgical trauma to the patients.

The mortality rate of IVH patients treated with endoscopic surgery at 1 year ranges from 10 to 30%, which is almost equivalent to the efficacy of EVD [1, 19, 20]. Several studies have shown that endoscopic surgery for IVH evacuation (with EVD) has advantages over EVD alone [1, 18], which revealed that the incidence of postoperative hydrocephalus and the dependency for ventricular-peritoneal shunt surgery postoperatively is lower in endoscopic group. The following factors might also be of great importance for improving patients’ outcome: (1) the endoscopic surgery could remove hematoma and improve cerebral perfusion quickly, reducing extrusion effects on critical cerebral structures and neurotoxic effects of the hematoma decomposition products; (2) neurosurgeons could evacuate the hematoma under direct vision, which increases the hematoma clearance rate and reduces the probability of postoperative rebleeding; and (3) the drainage catheter could be placed under direct view, improving placement accuracy and avoiding damage to the choroid plexus. However, the cases of most current research are small and all of them are retrospective studies. There are also no such clinical trials registered, which means there is no high-level evidence to verify the effect of endoscopic treatment for IVH because of the small sample size and retrospective analysis of the reported studies.

Based on this, we intend to conduct a randomized, controlled, multi-center clinical trial to compare the prognosis of patients who undergo endoscopic IVH evacuation surgery versus those who undergo external ventricular drainage for moderate to severe IVH.

## Methods

### Aim and objectives

Due to the insufficient evidence of endoscopic treatment for moderate to severe IVH, the aim of the study is to testify the efficacy of endoscopic IVH evacuation surgery compared with EVD.

### Study design

**Fig. 1:**
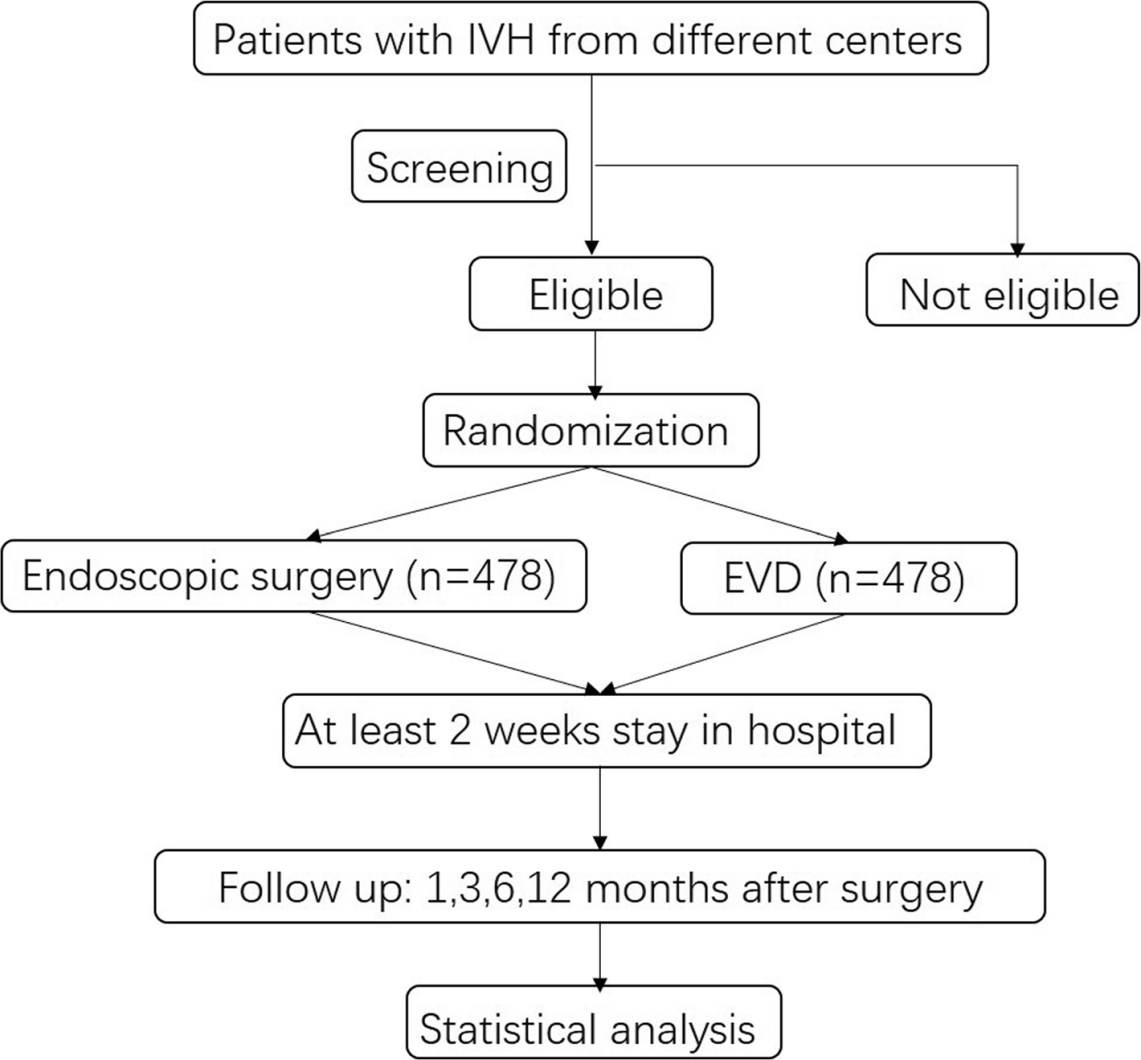
A flowchart of the study design

The study is a prospective, multicenter, randomized, controlled trial. A flowchart is shown in Fig. 1. Patients with IVH will be randomly assigned (1:1) to the endoscopic group and the EVD group.

The Standard Protocol Items: Recommendations for Interventional Trials (SPIRIT) checklist is provided in Additional file 1.

### Setting

The study will be performed at 4 neurosurgical centers in China (Jinling Hospital, Inner Mongolia People’s Hospital, The First Affiliated Hospital of Lanzhou University, and Shengjing Hospital Affiliated of China Medical University). The organizer has set up an expert panel to ensure that each center has the proper surgical technique.

First, the center applying to participate in the study needs to provide at least two videos of unedited endoscopic IVH evacuation surgery and EVD, respectively, and provide clinical information of at least 5 cases performed with endoscopic IVH evacuation surgery and EVD (including preoperative imaging data, surgical records, and postoperative imaging data). The expert panel will judge whether the applicant has the qualification to participate in the study based on the above information.

### Inclusion and exclusion criteria

#### Inclusion criteria

1. Patients of either sex, 18–70 years of age.
2. Clinical manifestation as moderate to severe IVH, such as severe headache, hemiplegia, aphasia, or unconsciousness.
3. Disease onset within 24 h.
4. Graeb scale > 4.
5. Glasgow Coma Scale < 13.
6. CT scan showing the existence of hemorrhage breaking into the ventricles or primary intraventricular hemorrhage, and the bleeding volume being larger than 50% of the volume of the lateral ventricle.

#### Exclusion criteria

1. Age < 18 years or > 70 years.
2. Patients with a history of severe cardiopulmonary dysfunction (NYHA level III or IV), chronic kidney disease (GFR < 60 ml/(min*1.73m^2^)), blood disorders (anemia (Hb < 90 g), hemophilia, myelodysplastic syndromes), cancer.
3. Patients who have any severe pre-existing physical or mental disabilities or comorbidities (palsy, dementia) that will interfere with the assessment of the outcome.
4. CT scan showing the existence of cerebellar hemorrhage or brain stem hemorrhage.
5. Cerebrovascular diseases detected by CTA/MRA/MRV/DSA examinations (choosing 1 or 2 examinations), such as aneurysm and cerebrovascular malformation.
6. CT scan suggesting the presence of brain tumors.
7. Patients with a history of coagulopathy or long-term use of anticoagulants.

### Recruitment and consent

The patients will be screened by experienced neurosurgeons in each center according to the inclusion and exclusion criteria. The surgeons should help patients understand the risks of two treatment strategies and written consent will be obtained from them. If the patients screened for this trial are unconscious or mentally impaired due to the stroke at admission, informed consent of their relatives will be obtained instead and consent of the patients will be obtained if they regain consciousness after treatment. If patients are incapacitated and their relatives cannot be located, these people will not be included in this trial. Any individual with a direct relationship to the researchers, such as close relatives or staff of the hospital, will not be included.

### Randomization

We adopt a central randomization method based on the mobile client randomization tool “Randomization Allocation Tool” (RAT) to achieve random assignment of patients into two groups at a 1:1 ratio. If the subject is qualified for the trial and signs the informed consent form, the investigator authorized by each center can input the relevant information (e.g., age, gender, the bleeding volume, scores of Graeb scale, and GCS) of the corresponding subject in the mobile phone client. After the administrator approves the confirmation, the assigned group is immediately fed back to the researcher’s mobile phone, and the researcher will perform the prescribed surgical treatment according to the specified group. Possible facts that may impact the outcomes of the patients, such as age, gender, scores of Graeb scale, and GCS, will be considered by a minimization random allocation system.

### Treatment

#### Endoscopic group

The patient in the endoscopic group will be placed in the supine position and under general anesthesia. Endoscopy is performed using a rigid endoscope (Karl Storz, German). The side with more hemorrhage will be taken as the surgical side and a transverse incision of about 3 cm in length will be performed 1–2 cm in front of the coronal suture, 2–3 cm beside the midline. Then, a bone window with a diameter of 2–3 cm will be made and we will cross-incise the dura mater. Next, the surgeons insert the endoport (Vycor Medical, USA) into the lateral ventricle, which will become the endoscopic work channel. The hematoma will be removed by a technique using irrigation and aspiration. After exposing the choroid plexus of the lateral ventricle, surgeons should strive to clear the hematoma in the third ventricle through the interventricular orifice. At the same time, the blood clots will be removed with grasping forceps and if there is bleeding, bipolar coagulation can be used to stop bleeding. During the operation, the veins, choroid plexus, and ventricular wall should be carefully protected and blood clots that are closely adhering to the choroid plexus are not required to be completely removed. The third ventriculostomy and pellucid septostomy should be performed if possible. The ventricular drainage catheter will be placed on the surgical side. The dura and skin will be closed in a routine manner. An immediate postoperative CT scan will be performed to assess the residual hematoma. After 6 h postoperatively, we will administer 20,000 U urokinase with 5 ml saline every 8 h through the catheter, and the catheter will be clamped for 1 h to allow drug–clot interaction and then reopened to allow for gravitational drainage. Subsequent CT scans will be done for any safety concern or every 24 h. Administration of urokinase will be stopped when the CT scans show that the circulation of cerebrospinal fluid is unobstructed. When CT scans show that the intracerebral hematoma is significantly reduced and the circulation of cerebrospinal fluid is unobstructed, the catheter shall be clamped for 24 h before removing the catheter. If there is no acute intracranial pressure increase, the catheter can be removed then.

#### EVD group

The patient in the EVD group will be placed in the supine position and under general anesthesia. Bilateral external ventricular drainage will be performed regularly and unilateral drainage will be performed only when the other lateral ventricular has little hematoma and the circulation of cerebrospinal fluid is unobstructed. Surgeons usually select the point 1–2 cm before the coronal suture of the bleeding side and 2–3 cm next to the midline as the puncture point and puncture inwardly along the plane of the puncture point and the line of the ear. The surgeons will use a soft catheter with a guide needle to puncture in depth of about 5 cm and then pull out the guide needle. The next step is to fix the drainage catheter and suture the scalp incision. Postoperative CT will be done immediately to confirm the positioning of the soft catheter and stability of the hematoma. After at least 6 h postoperatively, we will administer 20,000 U urokinase with 5 ml saline every 8 h, and the catheter will be clamped for 1 h to allow drug–clot interaction and then reopened to allow for gravitational drainage. Subsequent CT scans will be done for any safety concern or every 24 h. Administration of urokinase will be stopped when the CT scans show that the circulation of cerebrospinal fluid is unobstructed. When CT scans show that the intracerebral hematoma is significantly reduced and the circulation of cerebrospinal fluid is unobstructed, the catheter shall be clamped for 24 h before removing the catheter. If there is no acute intracranial pressure increase, the catheter can be removed then.

### Outcome measures

The primary outcome is the survival rate of patients at 12 months after surgery.

In addition, secondary outcome measures include treatment-related morbidity, as evaluated by the following items:

- Overall survival (time to death after surgery).
- Modified Rankin score (preoperational, 1 month, 3 months, 6 months, 12 months).
- Proportion of patients who need ventricular-peritoneal shunt after surgery (1 month, 3 months, 6 months, 12 months).
- Incidence of postoperative hydrocephalus (1 month, 3 months, 6 months, 12 months).
- Postoperative intracranial infection rate (1 month, 3 months, 6 months, 12 months).
- Hospital length of stay.
- Hospitalization expenses (costs spent during treatment for IVH in hospital).

The relevant data will be collected by two data entry staff independently.

The study will include a follow-up period of 12 months. Patients will stay in the hospital for at least 2 weeks postoperatively to complete the required assessments (e.g., GSC and Graeb scale, blood and urine tests, brain CT and MRI, CTA/MRA/MRV/DSA) which are shown in Table 1. The final study follow-up is scheduled at 12 months after surgery. The patients will be reminded to return to the hospital by phone calls at 1 month, 3 months, 6 months, and 12 months after surgery to perform associated clinical examinations and tests (e.g., blood and urine tests, brain CT and MRI, modified Rankin Scale, records of the complications and survival state).

**Table 1:** Enrollment and assessments in the trial

Item	Screening period	Follow-up							
		Operation day	1 day after the operation	7 days after the operation	2 weeks after the operation	1 month after the operation	3 months after the operation	6 months after the operation	12 months after operation
**Informed consent**	X								
**Collect demographic data**	X								
**Collect medical history**	X								
**Physical examination**	X	X	X	X	X	X	X	X	X
**Routine blood examination**	X	X	X	X	X	X	X	X	X
**Blood biochemical examination**	X	X	X	X	X	X	X	X	X
**Routine urine test**	X		X	X	X	X	X	X	X
**Brain CT scan**	X	X	X	X	X	X	X	X	X
**Brain MRI**	X					X	X	X	X
**CTA/MRA/MRV/DSA (choose 1 or 2 items)**	X								
**HIV, HBV, HCV screening**	X								
**Graeb score**	X	X	X	X	X	X			
**Glasgow Coma Scale**	X	X	X	X	X	X			
**Inclusion/exclusion criteria**	X								
**Randomization**	X								
**Survival rate at 12 months postoperatively**									X
**Modified Rankin Scale**		X	X	X	X	X	X	X	X
**Postoperative patient survival (OS)**		X	X	X	X	X	X	X	X
**Postoperative complications**		X	X	X	X	X	X	X	X

### Blinding

Due to the different surgical strategies of the two groups, no data could be collected blinded. The statistical analysis will be performed by specialized persons who are blinded as to the allocated intervention.

### Data collection and data management

Case report forms (CRFs) and clinical reports will be collected as the main data source in this trial. According to the regulations, two data entry staff will input the contents into the database independently as double copies. The database will be locked and transferred to the quality control group through the data management network after a data consistency check and a data quality audit are completed.

### Sample size

The sample size calculation results in a requirement of 956 patients. For EVD, the mortality rate is 23% in a meta-analysis of 4 randomized and 8 observational studies of patients with IVH [11]. In the CLEAR III trial, the mortality rate of the IVH patients treated with EVD plus rtPA is 18% at 180 days [13]. And the reported mortality rate of IVH patients treated with endoscopic surgery at 1 year ranges from 10 to 30% [1, 19, 20]. Based on previous studies and our own experience, we assume a 1-year overall survival rate of 70% (mortality rate of 30%) in both the reference group and the new surgical group. We plan for the trial to show non-inferiority of a new surgical method to the conventional method with a hazard ratio (HR) margin of 1.3 [21, 22]. The principle of the sample size calculation of a non-inferiority trial is different from that of a superiority trial where you want to demonstrate that one treatment or intervention is better than another. In the context of non-inferiority trials, the non-inferiority limit is used to calculate the sample size. The percentage for those on the experimental treatment is no worse than the percentage for those on the control treatment by the non-inferiority limit. In our study, the non-inferiority limit of the hazards ratio (HR) is set to 1.3. Accounting for a potential dropout rate of 5%, 956 patients (478 in each group) are needed to be enrolled, which ensured a power of 80%.

### Statistical analysis

All statistical analyses in the study will be carried out by an analyst who is blinded as to the allocated intervention. The primary efficacy will be compared by an intention-to-treat analysis including all randomized patients.

Regarding the primary outcome (the 12-month survival rate), the Cox regression model method will be used to calculate the hazards ratio and the 95% confidence interval (CI). The non-inferiority of the new surgical method and the conventional method is determined according to whether the upper limit of the 95% CI of the HR is less than 1.3. If the upper limit of the 95% CI is less than 1.3, the non-inferiority is accepted. Otherwise, a non-inferiority conclusion cannot be reached.

For the secondary outcomes, the OS curves will be estimated using the Kaplan–Meier method. Continuous variables will be assessed for normality and equality of variances between groups. Discrete variables will be summarized by frequencies/proportions. For continuous variables, analysis of variance and/or regression will be used, where appropriate (hospital length of stay, hospitalization expenses). The comparison of the two groups with respect to frequencies/proportions will be performed using the *χ*^2^ test and, if necessary, Fisher’s test (proportion of patients who need ventricular-peritoneal shunt after surgery, incidence of postoperative hydrocephalus, postoperative intracranial infection rate). The ranked data of the two groups will be compared using the Wilcoxon rank-sum test (mRS).

At the annual monitoring meeting of the Data and Safety Monitoring Board, the treatment effectiveness (the 12-month survival rate) will be compared between the two groups. If one group has significantly more hazards than the other one (upper limit of the 95% CI of the hazards ratio is more than 1.3), the trial will be terminated.

### Trial oversight and monitoring

The study will be reviewed and monitored annually by the Ethics Committee of the Jinling Hospital and the Data and Safety Monitoring Board whose members are from each center to ensure the safety of the participants and the validity of the data.

Termination of the trial may occur for the following reasons (determined by the Ethics Committee of the Jinling Hospital during their annual meeting):

1. Major mistakes are found in the trial protocol, making it difficult to evaluate the efficacy of the method.
2. The trial has a major deviation in implementation and thus is difficult to continue.
3. The sponsor requests to terminate the trial because of a lack of funding or poor management.

## Discussion

This study is a randomized controlled trial designed for patients with moderate to severe IVH. It aims to compare a relatively new surgical procedure (endoscopic IVH evacuation surgery) with the standard treatment (EVD). As with all surgical clinical trials, timing of the trial is crucial. If the trial is conducted too early, the new technique may still undergo too many modifications to allow application of a standardized procedure. In addition, recruitment might be too slow and the trial might turn out to be unfeasible. In contrast, if the trial is left too late, the new technique may become part of the mainstream treatment without adequate proof of its equivalence. In that case, too many patients might refuse randomization and the trial could also become unfeasible. Currently, the interest among endoscopy is strong and investigators are eager to enroll patients.

In summary, the available evidence suggests that endoscopic IVH evacuation surgery may be a valid alternative surgery to EVD with equal OS for patients diagnosed with moderate to severe IVH. This trial is aimed to provide high-quality evidence to support this hypothesis.

## Trial status

The trial was reregistered on July 25, 2019, at ClinicalTrials.gov and recruitment will begin in 2020. The programmed completion date for the recruitment is September 31, 2023, and will include a follow-up period of 12 months which means 4 years are needed to finish the trial. The trial is now in the recruitment stage. The protocol version number is V2.0 (April 23, 2019).

## Supplementary information

**Additional file 1.** SPIRIT 2013 Checklist: Recommended items to address in a clinical trial protocol and related documents.

## Data Availability

The data are currently unavailable.

## Abbreviations

IVH: Intraventricular hemorrhage
ICH: Intracranial hemorrhage
EVD: External ventricular drainage
STICH: Surgical Trial in Intracerebral Haemorrhage
AHA/ASA: American Heart Association/American Stroke Association
rtPA: Recombinant tissue-type plasminogen activator
CLEAR: Clot Lysis: Evaluating Accelerated Resolution of Intraventricular Hemorrhage
CTA/MRA/MRV/DSA: Computed tomography angiography/magnetic resonance angiography/magnetic resonance venogram/digital subtraction angiography
SPIRIT: The Standard Protocol Items: Recommendations for Interventional Trials
RAT: Randomization Allocation Tool
CT: Computed tomography
OS: Overall survival
CI: Confidence interval
